# Mapping the montane cloud forest of Taiwan using 12 year MODIS-derived ground fog frequency data

**DOI:** 10.1371/journal.pone.0172663

**Published:** 2017-02-28

**Authors:** Hans Martin Schulz, Ching-Feng Li, Boris Thies, Shih-Chieh Chang, Jörg Bendix

**Affiliations:** 1 Laboratory for Climatology and Remote Sensing, Philipps-Universität Marburg, Marburg, Germany; 2 School of Forestry and Resource Conservation, National Taiwan University, Taipei, Taiwan; 3 Department of Natural Resources and Environmental Studies, National Dong Hwa University, Hualien, Taiwan; Pacific Northwest National Laboratory, UNITED STATES

## Abstract

Up until now montane cloud forest (MCF) in Taiwan has only been mapped for selected areas of vegetation plots. This paper presents the first comprehensive map of MCF distribution for the entire island. For its creation, a Random Forest model was trained with vegetation plots from the National Vegetation Database of Taiwan that were classified as “MCF” or “non-MCF”. This model predicted the distribution of MCF from a raster data set of parameters derived from a digital elevation model (DEM), Landsat channels and texture measures derived from them as well as ground fog frequency data derived from the Moderate Resolution Imaging Spectroradiometer. While the DEM parameters and Landsat data predicted much of the cloud forest’s location, local deviations in the altitudinal distribution of MCF linked to the monsoonal influence as well as the Massenerhebung effect (causing MCF in atypically low altitudes) were only captured once fog frequency data was included. Therefore, our study suggests that ground fog data are most useful for accurately mapping MCF.

## Introduction

Montane cloud forest (MCF) is characterized by significant precipitation input from the canopy interception of frequently or persistently occurring foggy conditions at ground (tree) level [1]. On a regional scale, most endemic species can be found in MCF areas due to unique hydrological processes and how they interact with the biological communities [2, 3]. At the same time, MCF has been recognized as one of the world’s most endangered ecosystems [4, 5]. Climate observations from the past decade show that MCF areas suffer from a decreasing trend in ground fog occurrence that is likely related to global warming [6–11]. Further, the habitats of this ecosystem are heavily impacted by anthropogenic influences such as deforestation for timber harvesting and agriculture [5, 12–15]. Hence, worldwide efforts to reliably map the actual distribution of MCF are urgently required for the purpose of natural resources management.

Subtropical MCF in Taiwan has never been comprehensively mapped. It is, for example, absent from a map of global MCF [5] distributed by the United Nations Environment Programme (UNEP). Today, the National Vegetation Database of Taiwan provides the most reliable data about its extent. However, the data are spatially restricted to vegetation plots distributed across the island (cf. Sect. *Training data for the MCF conditions map*). Therefore, our study aims to map the entire distribution of Taiwanese MCF.

Simple MCF mapping approaches (e.g., [16] or the UNEP approach) use altitude as the predicting variable for MCF occurrence as it is a proxy for climatic (temperature, rainfall, and particularly ground fog frequency) and edaphic (soil water status and acidity) factors that are associated with the occurrence of MCF [17]. Altitude is not a perfect proxy, however. One important reason for this in Taiwan is the influence of the East Asian Monsoon which creates local deviations in the altitudinal distribution of ground fog occurrence. The Massenerhebung effect also seems to have similar impacts. Both have been considered as an explanation for the occurrence of MCF at atypically low altitudes (cf. Sect. Study area). An approach used by Mulligan and Burke [18] to map MCF in the global tropics could not capture these effects in the tropical south of Taiwan. The authors mapped MCF based on ground fog frequency modelled from reanalysis data [19–21], the resolution of which is too low to reproduce the influence either of the monsoon or the Massenerhebung effect. To investigate the suitability of remote sensing methods for MCF mapping, Nair et al. [22] compared a ground fog frequency map derived from 1-km Moderate Resolution Imaging Spectroradiometer (MODIS) data to 13 points from the UNEP-WCMC cloud forest locations database [23] in Costa Rica, southern Nicaragua, and northern Panama. They were able to capture local leeward effects and found a connection between high frequency of ground fog and MCF occurrence. This suggests that local altitudinal deviations of ground fog occurrence can be mapped using MODIS data. Thies et al. [24] and Wilson and Jetz [25] examined the relationship between MODIS-based 1-km cloud climatologies and MCF occurrence for Taiwan and for the global tropics (including the tropical south of Taiwan), respectively. While Thies et al. could not show a relationship between cloud frequency and MCF occurrence for most types of MCF, Wilson and Jetz created a global map of tropical MCF incorporating cloud frequency, inter-annual cloud variability, intra-annual cloud variability and elevation. Both studies mention that their approaches fail to separate clouds with and without ground contact, as MODIS cloud products do not include the height of the cloud base. Therefore, we present the first map of Taiwanese MCF distribution that incorporates remote sensing-based ground fog frequency data.

## Study area

Taiwan (21°85′—25°30′N, 120°00′—122°00′E, ∼ 36,000 km^2^) is a subtropical island in East Asia with a maximum elevation of 3952 m a.s.l. and more than 200 peaks above 3000 m a.s.l. Climatic variation in Taiwan is mostly correlated to altitude and monsoonal influence.

**Fig 1:**
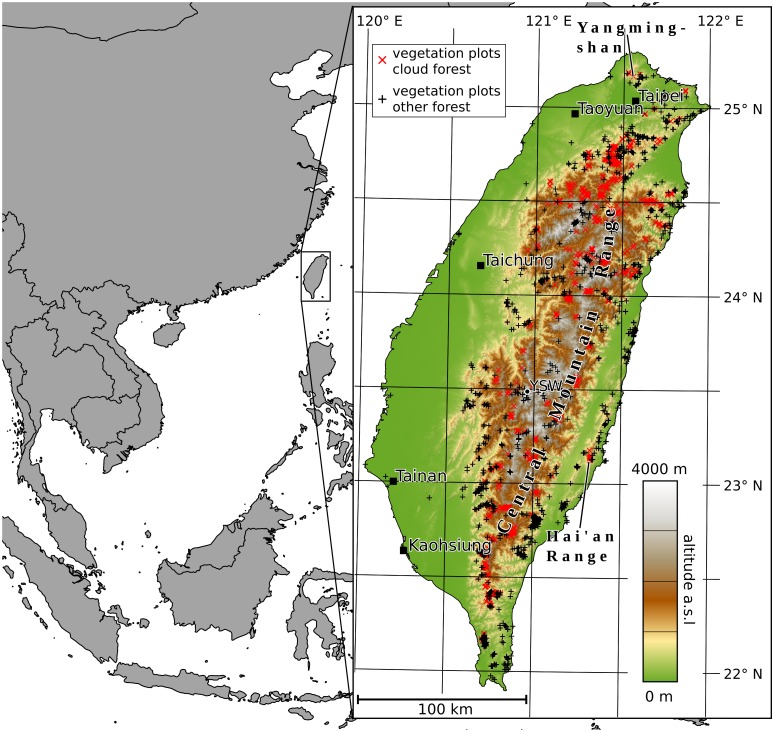
Topography and geographical location of Taiwan. The topography was derived from the ASTER GDEM 2 digital elevation model (cf. Sect. *Digital elevation model and related inputs*). The depicted vegetation plots are described in Sect. *Training data for the MCF conditions map*. Country borders were taken from OpenStreetMap [27].

Monthly mean temperatures range from -1.1°C in the Central Mountain Range (CMR) in January (measured at an altitude of around 3850 m a.s.l. by Yushan Weather Station; YSW in Fig 1) to about 29°C in the lowlands in July. Annual mean temperatures as low as 4.2°C can be observed in the mountains. In the lowlands, the annual mean temperatures are about 17°C in the north and 20°C in the south [26].

The summer monsoon between May and August approaches the island from the southwest, bringing, together with 3—4 typhoons per year, heavy rains to the entire island. The winter monsoon brings mild rainfall from the northeast direction to the windward areas of the island between September and April. Annual precipitation in the eastern lowlands exceeds 2000 mm, while the western part, which is less affected by the winter monsoon, receives less than 2000 mm. Most Taiwanese weather stations at altitudes higher than 1500 m a.s.l. measure annual precipitations greater than 2500 mm [28].

Forests in Taiwan can be classified into five vegetative zones based on local climate, which is primarily driven by altitude. At altitudes of ∼ 0—500 m a.s.l. and ∼ 500—1500 m a.s.l., respectively, the foothill zone and sub-montane zone are dominated by evergreen broad-leaved trees. At altitudes of ∼ 1500—2500 m a.s.l. the montane zone is characterized by frequent ground fog occurrence. It is dominated by either a mix of deciduous broad-leaved, coniferous, and evergreen broad-leaved forests or purely evergreen broad-leaved forests, both with characteristic features of MCF. Coniferous forests grow in the high-montane zone and subalpine zone at altitudes of ∼ 2500—3300 m a.s.l. and ∼ 3300—3700 m a.s.l., respectively. These zonal forests can be further classified into subtropical and tropical types. Northern Taiwanese flora belongs to the Holarctic Kingdom, while flora of the Paleotropical Kingdom can be found in the south. Azonal forests correspond less to the climatic influence (mostly related to altitude) than the zonal forests mentioned above. The distribution of azonal forests is related to non-climatic factors such as fire regime, landslides, human disturbance, and rocky outcrops [29].

**Fig 2:**
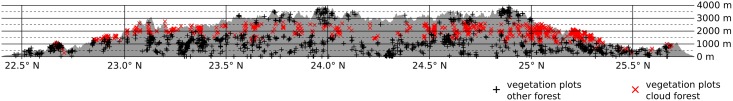
Altitudinal occurrence of MCF and non-MCF vegetation plots. The depicted vegetation plots are described in Sect. *Training data for the MCF conditions map*.

As the plots from the National Vegetation Database of Taiwan show, large areas of MCF can be found outside of the 1500 to 2500 m a.s.l. range. The distribution reaches altitudes as low as 1000 m a.s.l. in the northern and southern parts of the CMR. Isolated occurrences (particularly in the north) can even be found below 500 m a.s.l. (cf. Fig 2). Two factors can be assumed to be responsible for this distribution:

- The winter monsoon brings heavy fog to the windward parts of the island [3, 30]. This was linked to MCF occurrence at atypically low altitudes in northeastern Taiwan (cf. Figs 1 and 2)[3, 29].
- In isolated mountain terrain—especially if it is located near the ocean—the Massenerhebung effect causes a contraction/lowering of vegetation zones. This is particularly the case for MCF as condensation levels (and thus the altitude of frequent cloud immersion of the terrain) are shifted downwards. This is explained by a steep temperature lapse rate of isolated mountain terrain and a high water vapor concentration in the atmosphere around isolated coastal mountains [6, 31–33]. As the mountainous terrain is more isolated to the north and south of the CMR than in the center of Taiwan, the Massenerhebung effect is considered an explanation for lowered altitudes of the vegetation zones here [34].

## Data and methods

### Overall methodology

The map of Taiwanese MCF presented in this study was created by the intersection of two binary maps:

- A map of MCF conditions.
- A forest map (Sect. *Creation of the forest map*).

The usage of a machine learning approach allows additional inputs (besides ground fog frequency) to be incorporated. A Random Forest classifier [35] (implemented in the R [36] package “randomForest” [37]) was used to create the map of MCF conditions. Several studies have already shown the effectiveness of this ensemble machine learning method in vegetation mapping using remotely sensed data [38–40]. The Random Forest model was trained to distinguish the classes “MCF” and “non-MCF” using training samples extracted from different raster inputs with a resolution of 250 m per pixel. These inputs were monthly ground fog frequency maps (cf. Sect. *Ground fog frequency inputs*), different DEM parameters (Sect. *Digital elevation model and related inputs*) as well as mosaics of the Landsat 7 solar bands (Sect. *Landsat channels*) and texture measures calculated from them (Sect. *Geostatistical texture features*). The positions from which training data were extracted were given by a point data set, which contains information about the presence or absence of MCF (cf. Sect. *Training data for the MCF conditions map*). After training, the number of input variables was reduced in order to avoid the “curse of dimensionality” [41] and increase the quality classification. This was done using the recursive feature elimination method implemented in the R package “caret” [42]. The reduced inputs were used to predict the occurrence of MCF conditions in the 250 m resolution of the input raster data.

Pixels determined to have MCF conditions are not necessarily covered by forest, since the data points used to create the map of MCF conditions do not distinguish between forested and non-forested areas. Since cloud forest can only exist where there is indeed forest, the map of MCF conditions was combined with the forest map.

### Ground fog frequency inputs

Data from the Moderate-resolution Imaging Spectroradiometer (MODIS) onboard the near-polar orbiting satellites Terra (daytime Taiwan overflight ∼ 9:30—11:30 UTC + 8) and Aqua (daytime Taiwan overflight ∼ 12:30—14:30 UTC + 8) were used to create the ground fog frequency maps. Bands 1 and 2 of the instrument each have a resolution of 250 m per pixel, which produce a detailed image of ground fog and the shape of its extent as restricted by complex terrain. The distribution of MCF is highly dependent on altitude and, in mountainous areas, a coarse resolution implies a high altitudinal span covered by each pixel. Therefore, MODIS was chosen instead of the geostationary satellite Himawari 7, which covers Taiwan with a spatial resolution of up to 1000 m and a temporal sampling rate of 30 minutes. Although the newer Himawari 8 has a spatial resolution of up to 500 m, this data was disregarded as the satellite has only been in service since July 2015. This active service period is too short for the creation of meaningful ground fog frequency maps.

Common remote sensing-based ground fog detection schemes rely on assumptions regarding the microphysical properties of fog clouds, their vertical distribution within the cloud, and a certain relationship between the cloud top temperature and the cloud top height. As they are based on observations of radiation fog, which is common in temperate latitudes, those assumptions are not suited for fog in Taiwan, which is mostly orographic. Schulz et al. [43] therefore developed the algorithm *Detection of Ground Fog in Mountainous Areas* (DOGMA), which is custom tailored and validated for ground fog detection in the mountains of Taiwan. DOGMA detects fog in MODIS daytime scenes based on the DEM and the MODIS MOD 06 cloud optical thickness product (nighttime scenes can not be processed for the lack of a nighttime optical thickness input) using a statistical approach. The method sharpens the MODIS input data using the high-resolution MODIS bands 1 and 2 resulting in cloud masks with a resolution of 250 m.

DOGMA was used to create ground fog masks for every MODIS daytime scene covering Taiwan between 1 January 2003 and 31 December 2014. These fog masks were used to create ground fog frequency maps for the whole year as well as for each individual month, in order to capture the influence of the seasonality of ground fog occurrence.

### Digital elevation model and related inputs

Other factors that are linked to the distribution of MCF (temperature, rainfall, soil water status and acidity) are not available as high-resolution raster inputs. Therefore, altitude was included in the machine learning approach as a proxy for those variables (cf. Sect. Introduction). It was taken from the ASTER GDEM 2 (property of METI and NASA) distributed via the USGS global data explorer [44]. ASTER GDEM 2 originally has a resolution of 1 arcsecond (∼ 30 m) and was resampled to the 250 m resolution of DOGMA for our machine learning approach.

Li et al. [3] successfully used an ordinal classification of the topography of Taiwan (1 = ridge, 2 = upper slope, 3 = middle slope, 4 = lower slope, 5 = valley, 6 = plain) as a predictor variable for the floristic composition of cloud forests in Taiwan. This classification system was used as a proxy for soil water availability and light input. Several vegetation plots were manually classified by Li et al., but this is not expedient for the creation of an input file for all of Taiwan. Therefore, several quantitative inputs that essentially contain the same information as the ordinal classification by Li et al. were calculated from the original 30 m version of the ASTER GDEM 2 and transferred to the 250 m resolution afterwards:

- The **sky view factor** is related to topographic shading [45]. Its values are high on ridges and low in valleys.
- The **slope** corresponds to the steepness of the terrain. It is low on valley bottoms and plains as well as on mountain tops. It is high on valley flanks.
- The **distance to the ridge** gives information about the segment of a slope in which a pixel is located. In order to calculate the distance to the ridge, terrain was divided into 4 main classes of its aspect using k-means clustering. For each entity of spatially connected pixels of the same class, the vertical distance to the highest pixel of that entity was calculated.
- The **distance to the valley bottom** is calculated in the same way as the distance to the ridge. Here, the vertical distance to the lowest pixel of an entity is calculated instead of the distance to the highest pixel.

### Landsat channels

Landsat 7 Enhanced Thematic Mapper Plus (ETM+) visible and shortwave infrared bands 1, 2, 3, 4, 5 and 7 were used in the machine learning approach to account for spectral characteristics of MCF. Mosaics covering the whole area of Taiwan were compiled from 25 relatively cloudless ETM+ scenes captured between 1999 and 2003 (newer imagery has data gaps due to the failure of the ETM+ scan line corrector in May 2003).

Effects of atmospheric absorption, reflection, and scattering were removed from the Landsat 7 bands using the Second Simulation of a Satellite Signal in the Solar Spectrum code (6S code [46]) modified as described in [47]. The modified version is able to cope with a wide range of terrain altitudes in a scene. The Landsat bands were topographically corrected using a modified version of an approach suggested by Teillet et al. [48]. We masked out clouds as described in [49]. Terrain shadows were masked out by calculating their position based on the ASTER GDEM 2 and the sun azimuth and zenith.

After the atmospheric and topographic correction, bands 1, 2, 3, 4, 5 and 7 of all Landsat scenes were each stitched together in a cloud and shadow free mosaic. The mosaics are publicly available under doi:10.5678/LCRS/DAT.283. For the usage in the machine learning approach, the mosaics were resampled to the 250 m resolution of the other machine learning inputs. Texture measures were calculated from the mosaics of each band in the original 30 m Landsat resolution.

### Geostatistical texture features

Geostatistical texture features characterize textures based on the relationship between the similarity and distance between pixels of remotely sensed images [50]. Various studies [39, 51] have shown that their usage can enhance the quality of forest classifications. They could also be useful for MCF mapping as trees often have a reduced stature under MCF conditions [1], which may affect the texture of Landsat bands.

We created texture images of the Landsat composites using the texture features variogram, madogram, rodogram, cross variogram, and pseudo cross-variogram [50, 51]. While the variogram, madogram and rodogram compare pixels of the same raster input to each other, the cross variogram and the pseudo-cross variogram characterize the spatial variability based on pixels of different raster inputs. The variogram, madogram and rodogram texture parameters were calculated from all Landsat bands. The cross variogram and pseudo cross-variogram parameters were calculated from each possible combination of two different Landsat bands. Each texture input was calculated twice, using lag distances of 1 and 2 pixels and a window size of 7 pixels.

### Training data for the MCF conditions map

The data points used to train the Random Forest model for MCF conditions mapping were taken from the National Vegetation Database of Taiwan (Global Index of Vegetation-Plot Databases ID: AS-TW-001). Azonal forest types identified by Li et al. [29] were removed from the original data set because their presence or absence is not related to climatic factors such as the frequency of fog formation. The remaining 2367 points were classified into the categories “MCF” and “non-MCF” based on the composition of their woody species (trees and shrubs).

According to [29] there are four types of montane cloud forests in Taiwan: C2A03 *Chamaecyparis* montane mixed cloud forest, C2A04 *Fagus* montane deciduous broad-leaved cloud forest, C2A05 *Quercus* montane evergreen broad-leaved forest and C3A09 *Pasania-Elaeocarpus* montane evergreen broad-leaved forest. The first three types have a subtropical flora and the last one has a tropical flora. Each type is defined in [29] using a cocktail formula that requires the presence of certain species groups (and the absences of others) for a specific vegetation type [52–54].

In the current study, the MCF types C2A04 and C3A09 from [29] and species groups defined in [3] for the other montane cloud forest types were used. The latter definition was used because the study by [3] led to a better understanding of *Chamaecyparis* and *Quercus* cloud forest resulting in a better definition of these two types of MCF. Altogether, 834 plots were selected as MCF points in the training data set (cf. Fig 1). All other zonal forest plots were defined as non-MCF. From those, plots containing montane cloud forest species groups defined in [3] were further excluded to make these points purely represent forests uninfluenced by fog. In the end, there were 1533 points selected as non-MCF in the training data set (cf. Fig 1).

The classified data points are publicly available under doi:10.5678/LCRS/DAT.147.

### Creation of the forest map

The training samples described in the previous section only include vegetation plots. The locations of unforested areas needed to be known as well to create the forest map. Therefore, 408 training locations for two classes of point data (forest and non-forest) distributed over the whole island were manually set in a GIS using the Landsat layers presented in Sect. *Landsat channels* and (in cases where the class could not be unambiguously identified based on the Landsat data) Google Earth as well as Google Street View data as reference. A Random Forest model was trained based on those point locations to detect forest in the Landsat data. After a recursive feature elimination was performed, the model was used to delineate the forested areas of Taiwan.

### Methodology of validation

One advantage of Random Forest classifiers is that the same data can be used for training and for validation using an out-of-bag approach [55]. This was done for the map of MCF conditions as well as for the forest map. Using confusion matrices comparing the prediction results (“MCF”, “non-MCF” / “forest”, “non-forest”) with the training data set, the following statistical measures were calculated [56]:

- Proportion correct (PC): Fraction of the point data that was correctly predicted by the binary classification;
- Bias: Over- and underestimation (values above and below 1, respectively) of MCF / forest occurrence;
- Probability of detection (POD): Fraction of MCF / forest points that were correctly predicted as MCF / forest;
- Probability of false detection (POFD): Fraction of non-MCF / non-forest points that were falsely predicted as MCF / forest;
- False alarm rate (FAR): Fraction of points for which MCF / forest was predicted that were actually non-MCF / non-forest;
- Matthews correlation coefficient (MCC) [57]: A measure for the overall quality of a binary classification. 1 = perfect agreement between classification and point data; -1 = total disagreement between classification and point data.

## Results and discussion

### Ground fog frequency maps

**Fig 3:**
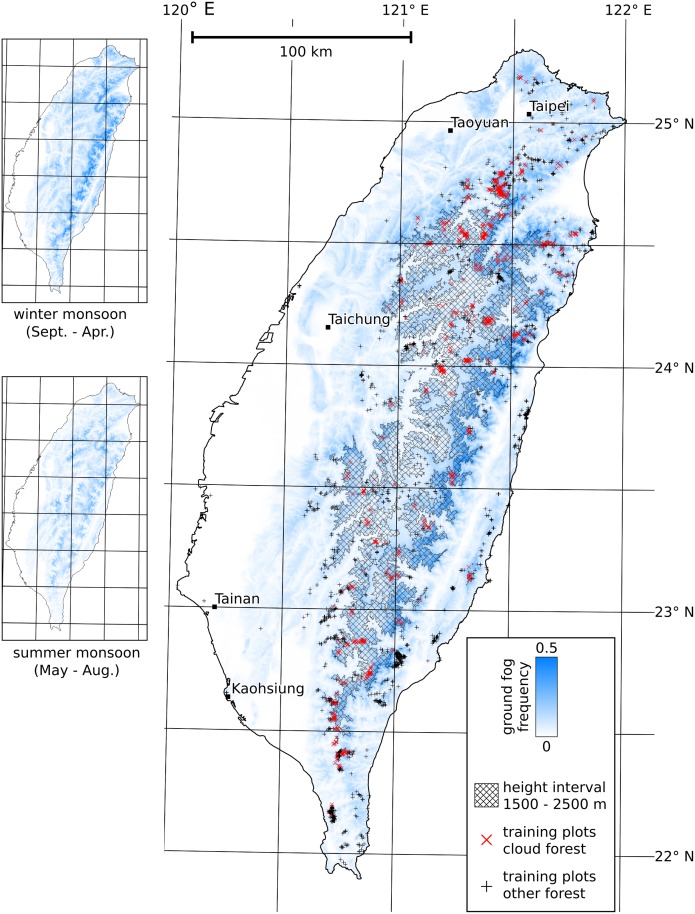
Ground fog frequency map calculated from MODIS data captured between 1 January 2003 and 31 December 2014.

Frequency maps created from the DOGMA ground fog masks (publicly available under doi: 10.5678/LCRS/DAT.145) are presented in Fig 3. The frequency of ground fog is generally high at altitudes between 1500 and 2500 m a.s.l. (cross-hatched area). This corresponds to the montane cloud zone with the highest MCF occurrence. Distinct local deviations in the altitudinal distribution of ground fog occurrence do exist, however. On the western slopes of the CMR, fog clearly forms less frequently than on the northern and eastern slopes. More fog occurs also at low altitudes on the northern and eastern slopes. This is particularly pronounced during the northeasterly winter monsoon between September and May. Due to the spatial pattern, it can be assumed that a causal link to the winter monsoon exists. In the summer months, fog generally appears less frequently and is more evenly distributed across the entire island. Areas with frequent occurrence of ground fog in isolated mountain terrain at altitudes below 1500 m a.s.l., e.g. in the Hai’an Range on the east coast or the Yangmingshan in northern Taiwan (cf. Fig 1), could be caused by the Massenerhebung effect.

As cloud frequency is independent from frequent cloud immersion of the terrain, it is—in contrast to the frequency of ground fog—hardly correlated to the altitude of the terrain. Therefore, the map of full-year ground fog frequency presented in the this study differs strongly from the cloud frequency maps presented by Thies et al. [24] and Wilson and Jetz [25] (cf. Sect. Introduction). In the latter, the cloud frequency between 1500 and 2500 m a.s.l. is, for example, similar to that in wide areas of the lowlands. However, the inter-annual cloud variability calculated by Wilson and Jetz shows low values on the northern and eastern slopes of the CMR in particular, as well as on the Hai’an Range, where our map reveals high ground fog frequency values. This must be caused by the spatial distribution of ground fog in Taiwan following yearly patterns more predictable than other clouds, most likely due to the influence of the winter monsoon.

### Maps of MCF conditions

Slope and sky view factor were the only DEM-based parameters excluded from the input data set by the recursive feature elimination. Bands 4 and 7 as well as all texture measures except six variants of the pseudo cross-variogram with different lag sizes and combinations of bands were removed from the Landsat-based inputs. The frequency input data set remained completely.

**Fig 4:**
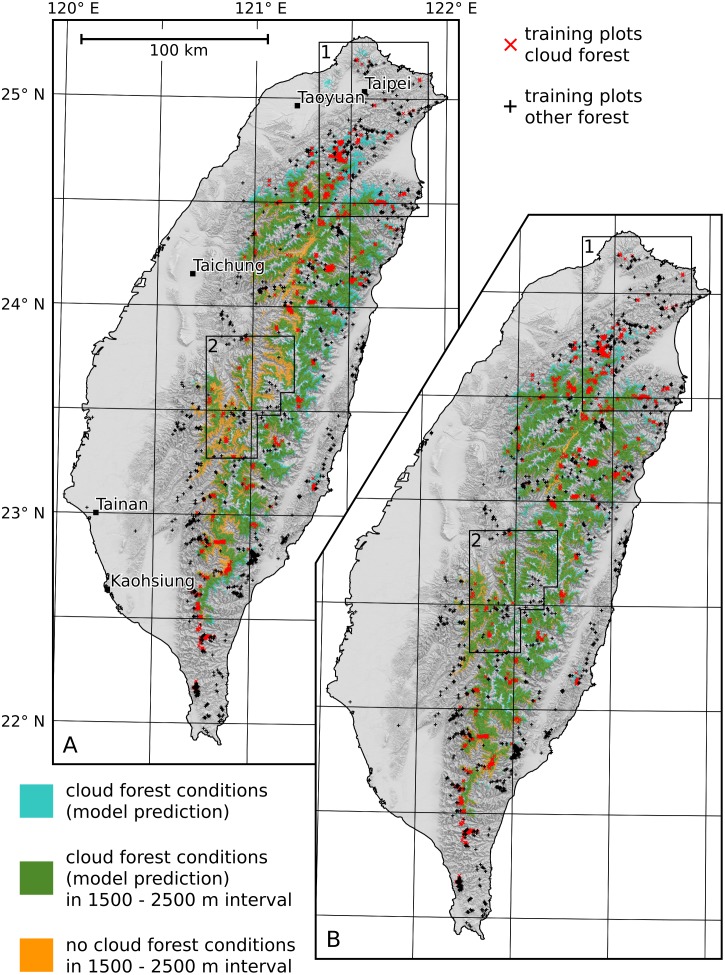
Maps of MCF conditions with (A) and without (B) the ground fog frequency being included.

The map of MCF conditions predicted by the Random Forest model based on the remaining inputs is presented in Fig 4A. It is publicly available under doi:10.5678/LCRS/DAT.278. A second map (Fig 4B) was created for comparison. The ground fog frequency inputs were excluded from the model inputs before the recursive feature elimination was conducted for this map.

Out-of-bag validation for both maps can be found in Table 1. The results reveal that both maps are of high quality. Map A has a slightly higher overall quality than map B in a direct comparison of the Matthews correlation coefficients and the proportion correct. The proportion of MCF plots correctly reproduced in the map (probability of detection) is also slightly higher in map A than for map B. The fraction of the plots for which MCF conditions were predicted although they are actually non-MCF plots (false alarm rate) is lower for map A. The fraction of non-MCF plots that were mistaken for MCF (probability of false detection) as well as the degree of underestimation of the area of MCF conditions (Bias) are the same for both maps.

**Table 1:** Validation results for MCF conditions maps A and B. The best value of each statistical parameter is written in bold type. TP = true positives; TN = true negatives; FP = false positives; FN = false negatives; MCC = Matthews correlation coefficient; PC = Proportion correct (PC); POD = Probability of detection; POFD = Probability of false detection; FAR = False alarm rate (FAR).

TP	TN	FP	FN	MCC	PC	Bias	POD	POFD	FAR	Validated map and height interval
765	1477	56	69	**0.88**	**0.95**	**0.99**	**0.92**	**0.04**	**0.07**	A
130	1259	28	50	**0.74**	**0.95**	**0.88**	**0.72**	**0.02**	**0.18**	A (<1500 m a.s.l.)
635	218	28	19	**0.87**	**0.95**	**1.01**	0.97	**0.11**	**0.04**	A (≥1500 m a.s.l.)
754	1466	67	80	0.86	0.94	**0.99**	0.90	**0.04**	0.08	B
112	1258	29	68	0.67	0.93	0.79	0.62	**0.02**	0.21	B (<1500 m a.s.l.)
642	208	38	12	0.86	0.94	1.04	**0.98**	0.15	0.06	B (≥1500 m a.s.l.)

In order to quantitatively assess the altitudinal dependency of the prediction quality, validation was performed a second time for vegetation plots at altitudes below 1500 m a.s.l. and above 1500 m a.s.l., respectively. The results are shown in Table 1. The validation based on plots above 1500 m a.s.l. shows a pattern that is similar to the validation result based on all plots. Here, however, the probability of detection is better in map B than in map A. Overall, the benefit of using frequency inputs seems insignificant at altitudes above 1500 m a.s.l. This is due to a relatively small transition zone from MCF to non-MCF conditions at an altitude of ∼ 2500 m a.s.l. (cf. Fig 2). Above this transition zone, MCF conditions hardly occur. Below this transition zone, almost all plots within the > 1500 m a.s.l. class are MCF. Therefore, DEM-related inputs alone are well suited to distinguish MCF plots from non-MCF plots here. As most of the MCF plots used in validation are located above 1500 m a.s.l., this is strongly reflected in the validation including all plots and explains similar validation results for both maps.

In the < 1500 m a.s.l. class, the overall prediction quality as measured by the Matthews correlation coefficient and the proportion correct is higher for map A. The difference in the Matthews correlation coefficient between map A and map B is much more distinct than in the validation including all plots. This is mostly due to a clearly higher probability of detection resulting in a higher (= better) Bias. In summary, frequency raster inputs clearly contribute to the correct prediction of MCF plots at low heights, where altitude is not a useful predictor. Significant visual differences between both maps reflect this finding. The area of MCF conditions in Fig 4B corresponds very well with the interval between 1500 and 2500 m a.s.l. There is no visible influence of local deviations in the altitudinal distribution of ground fog occurrence on the distribution of MCF. It is, however, captured in Fig 4A. More areas with MCF conditions can be found at altitudes below 1500 m a.s.l on the northern and eastern slopes of the CMR and in low areas in northern Taiwan (domain 1 in Fig 4 including Yangmingshan) as well as in the Hai’an Range. All of these areas are exposed to the winter monsoon and some of them have isolated mountain terrain. In contrast, MCF conditions can mainly be found at altitudes above 2000 m a.s.l. in the western part of the CMR in central Taiwan (domain 2 in Fig 4) that is less influenced by the winter monsoon.

In order to assess the explanatory power of the different input raster sets, our study independently validated the monthly ground fog frequencies, the DEM-based inputs as well as the Landsat bands and textures. The input raster sets were reduced in their dimensionality through recursive feature elimination and were each used to train a separate Random Forest model, which were then validated using the out-of-bag approach. The results are shown in Table 2. Most statistical parameters calculated from the frequency input raster set have the best values. As the monthly ground fog frequencies are better suited to create a map of MCF conditions than the DEM-based inputs, the relatively small advantage of the inclusion of ground fog frequency maps for the mappings of MCF conditions above 1500 m a.s.l. must be a result of the strong height dependence of ground fog frequency in these altitudes.

**Table 2:** Validation results for Random Forest models trained with different raster input sets. The best value of each statistical parameter is written in bold type. TP = true positives; TN = true negatives; FP = false positives; FN = false negatives; MCC = Matthews correlation coefficient; PC = Proportion correct (PC); POD = Probability of detection; POFD = Probability of false detection; FAR = False alarm rate (FAR).

TP	TN	FP	FN	MCC	PC	Bias	POD	POFD	FAR	used raster input set
723	1446	87	111	**0.82**	**0.92**	0.97	**0.87**	**0.06**	**0.11**	monthly ground fog frequency maps
719	1438	95	115	0.80	0.91	**0.98**	0.86	**0.06**	0.12	DEM-based inputs
686	1415	118	148	0.75	0.89	0.96	0.82	0.08	0.15	Landsat-based inputs

Clearly, the monthly ground fog frequency maps are not a perfect predictor for MCF conditions. Otherwise the Random Forest model based solely on frequency data would be as good as the models including more than one input raster set. This could be the result of MCF conditions depending on environmental factors other than ground fog frequency. Altitude, for example, is not only a proxy for the fog frequency but also for other climatic and edaphic parameters with impact on the floristic composition. Furthermore, the low temporal sampling rate (particularly the lack of nighttime scenes) of the satellite data used to create the ground fog frequency maps as well as imperfections in the fog detection algorithm (e.g., problems with clouds with an optical thickness greater than 40 [43]) result in flaws in the frequency data that reduce their explanatory power.

### The forest map and final MCF map

**Fig 5:**
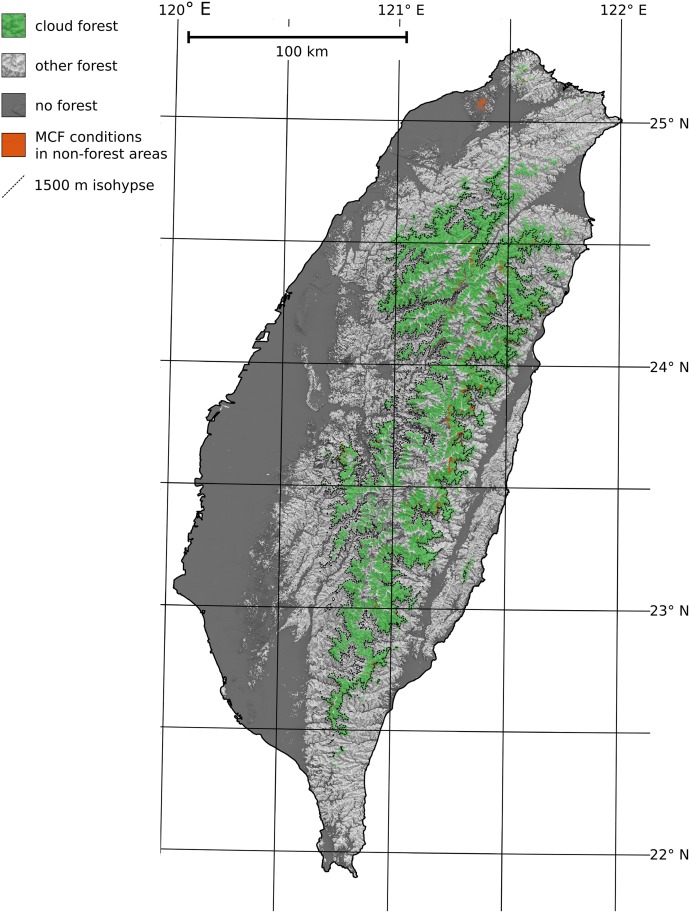
Final MCF map (green area), created from the combination of the MCF conditions map B (green and orange area) and the forest map (white and green area).

The forest map (publicly available under doi:10.5678/LCRS/DAT.278) is presented as the light grey and green area in Fig 5. The out-of-bag validation (223 true positives, 180 true negatives, 3 false positives, 3 false negatives) verifies that it is overall of very high quality (Matthews correlation coefficient = 0.97, proportion correct = 0.99). Almost all forest training samples were predicted correctly (probability of detection = 0.99) and almost none of the non-forest samples were mistaken for forest (probability of false detection = 0.02). In addition, nearly no sample for which forest was predicted was actually forest free (false alarm rate = 0.01). No systematic over- or underestimation occurred (Bias = 1.00).

The final MCF map—the intersection of the map of MCF conditions (Fig 4A) and the forest map—is presented as the green area in Fig 5. It is publicly available under doi:10.5678/LCRS/DAT.278. This map displays only pixels where both MCF conditions and actual forest occur. As MCF conditions mostly occur in the wooded center of Taiwan, the difference (orange area = pixels with MCF conditions but no forest occurrence) between the final MCF map and the map of MCF conditions is small. In general, relatively small features such as river beds or bedrock on steep slopes were removed from the map of MCF conditions. Only in the urban north of the island was a larger cluster of pixels removed.

Despite substantial differences in the frequency inputs and different spatial resolutions, the southern part of the MCF map presented in Fig 5 and the Taiwanese areas of tropical MCF mapped by Wilson and Jetz [25] mostly overlap. As the cloud frequency input used by Wilson and Jetz does distinguish the lower boundary of MCF conditions, it is reasonable to assume that the southern Taiwanese MCF was correctly mapped mostly due to the elevation input and the inter-annual cloud variability.

## Conclusion and outlook

This study produced the first comprehensive map of montane cloud forests in Taiwan. To the best of our knowledge, it is also the first study to successfully use a high-resolution ground fog frequency climatology in vegetation mapping. An MCF map based on DEM-derived parameters and Landsat data alone correctly locates much of the Taiwanese cloud forest. Including frequency data for ground fog in the machine learning inputs captures what we consider to be the influence of the Massenerhebung effect as well as the monsoonal impact on the island. This significantly enhances the detection of montane cloud forest at low altitudes.

As stated in Sect. *Maps of MCF conditions*, the low temporal sampling rate of MODIS data is an issue of the mapping approach presented in this study. MODIS was chosen over Himawari 7 due to its higher spatial resolution. Although the geostationary satellite Himawari 8 has a relatively high spatial resolution of 500 m per pixel and high temporal sampling rate of 10 minutes, it has only been in operational service above the western Pacific Ocean since July 2015. Once it has delivered sufficient data for the creation of meaningful ground fog frequency maps, it will help to further refine the mapping of MCF in Taiwan, e.g., by capturing intradiurnal variations of the ground fog frequency as described in [58]. Additional products could also be included in the machine learning approach, e.g. daily fog duration thanks to the high temporal sampling rate of Himawari 8. Also, the inclusion of further environmental parameters that can be derived from remotely sensed data might further enhance the correct delineation of Taiwanese MCF areas in future studies (Mulligan and Burke [18], for example, stressed the importance of the cloud cover frequency as a proxy for rainfall and the reduction of incoming radiation).
